# Age Is a Risk Factor for Gastroscopy-Assisted Capsule Endoscopy in Children

**DOI:** 10.5152/tjg.2023.22428

**Published:** 2024-01-01

**Authors:** Hongli Wang, Jing Xie, Lu Ren, Defeng Liang, Liya Xiong, Liying Liu, Wanfu Xu, Sitang Gong, Lanlan Geng, Peiyu Chen

**Affiliations:** 1 Department of Gastroenterology, Guangzhou Women and Children’s Medical Center, Guangzhou Medical University, Guangzhou, China; 2 Institute of Pediatrics, Guangzhou Women and Children’s Medical Center, Guangzhou Medical University, Guangzhou, China

**Keywords:** Children, capsule endoscopy, gastroscopy-assisted, small bowel transit time

## Abstract

**Background/Aims:**

The aim of this study was to explore the risk factors for the incidence of gastroscopy-assisted capsule endoscopy and the small bowel transit time in pediatric patients who underwent capsule endoscopy examination.

**Materials and Methods:**

A retrospective analysis was performed to analyze the clinical data collected from pediatric patients who underwent capsule endoscopy examination.

**Results:**

A total of 239 pediatric patients were enrolled in this study. About 196 (82.0%) patients completed the entire small bowel capsule endoscopy examination, while 3 (1.3%) patients were subjected to capsule retention. Only age, not gender, height, body weight, body mass index, chief complaint, and intestinal preparation medications, has been identified as a risk factor for the incidence of gastroscopy-assisted capsule endoscopy (*P *< .05) by multivariate logistic regression. Further analysis showed that the small bowel transit time in the self-swallowed group was shorter than that in the gastroscopy-assisted group, while no significant difference was obtained in other factors, including intestinal preparation medications, metoclopramide, and lesions in the small intestine, which did not significantly affect small bowel transit time compared with the corresponding control group (*P* > .05).

**Conclusion:**

A comprehensive assessment is required before performing capsule endoscopy, because age has been identified as a critical risk factor for the incidence of gastroscopy-assisted capsule endoscopy in pediatric patients.

**Main Points**

- Age has been identified as a risk factor for the incidence of gastroscopy-assisted capsule endoscopy (*P *< .05) by multivariate logistic regression.
- The small bowel transit time in the self-swallowed group was shorter than that in the gastroscopy-assisted group.
- A comprehensive assessment is required before performing capsule endoscopy, because age has been identified as a risk factor for the incidence of gastroscopy-assisted capsule endoscopy in pediatric patients.

## Introduction

The small intestine accounts for about 70%-75% length of the total digestive tract. Its anatomical position is inconstant, and most small intestinal diseases are concealed from the onset, which has been believed as the “blind area” in gastrointestinal tract examination. Traditional imaging technology, such as gastrointestinal radiography, abdominal ultrasonography, computed tomography (CT), magnetic resonance imaging (MRI), emission computed tomography (ECT), and magnetic resonance enterography (MRE), displayed intestinal wall thickening, edema, and space occupation with limited sensitivity and accuracy, and failed to inspect the intestinal cavity directly.^1^ Double-balloon enteroscopy can be utilized to inspect the intestinal cavity and perform the mucosal biopsy and endoscopic treatment under general anesthesia, while simultaneously being of high risk and poor tolerance in pediatric patients.^2^ It is difficult to operate as well as unable to examine the entire small bowel.

Interestingly, capsule endoscopy (CE) is a new non-invasive and efficient visualization method for the small intestine, avoiding potential damages of other inspections, such as ionizing radiation and trauma, which is suitable for pediatric gastrointestinal diseases under growing.^3,4^ However, in clinical practice, not all children are willing to cooperate with swallowing the CE or the CE cannot enter the small intestine for a long time after self-swallowing; in such cases, gastroscopy-assisted CE is required when necessary.

In this study, we reviewed the clinical data from 239 pediatric patients who underwent CE to explore the potential risk factors for gastroscopy-assisted CE, including gender, age, height, body weight, body mass index, chief complaint, and intestinal preparation medications. In addition, we also compared the SBTT between gastroscopy-assisted and self-swallowed CE groups and analyzed the influence factors of SBTT.

## Materials and Methods

### Patients and Ethical Statement

A total of 239 hospitalized pediatric patients characterized by small-bowel diseases in the Department of Gastroenterology in Guangzhou Women and Children’s Medical Center from April 2016 to December 2019 were collected in this study upon the Declaration of Helsinki as reflected in a prior approval approved by Medical Ethics Committee for Clinical Ethical Review of Guangzhou Women and Children’s Medical Center (Approval No. 2017111501). Informed consent was told by the caregiver of the child for his clinical records used, which are not publicly available; however, it could be available upon request.

### Inclusion and Exclusion Criteria

Children were suspected of small intestinal diseases. All patients underwent abdominal B-ultrasound examination. Among them, 212 patients received painless gastroscopy and colonoscopy simultaneously. Some patients performed the complete gastrointestinal radiography and abdominal CT, and no obvious obstruction or stenosis was found.

Exclusion criteria followed Chinese guidelines for the application of CE:^5^ (1) those who have no indications of operation or refuse any abdominal surgery; (2) those who have known or suspected of gastrointestinal obstruction, stenosis, or fistula; and (3) those who are carrying pacemakers or other electronic instruments implanted.

### Equipment

MiroCam CE system (IntroMedic Co. Ltd., Republic of Korea) was used, composed of a CE, an image recorder, and an image workstation. The capsule size is 10.8 mm × 24 mm, with weight of 3.25 g. This CE can observe intestine mucosa with a wide-angle greater than 170° and obtain 320 × 320 pixels high-definition images. This system employs E-field transmission technology, captures pictures at a rate of 3 frames/s, works continuously for at least 11 hours, and sends at least 120 000 photos to the image recorder.

### Preoperative Preparation

All patients took the liquid diet 3 days before the examination. The bowel preparation began 24 hours before the procedure: all the patients took polyethylene glycol electrolyte solution or 20% mannitol solution randomly until watery stools were discharged 3-4 times. Then they were fasted and allowed to drink the colorless beverage 12 hours before the operation. During the preparation period, the mental state of patients was closely monitored. Patients would receive intravenous fluid therapy symptomatically if hypoglycemia or dehydration was observed.

### Examination Procedures

Performance of CE examination in a standard procedure. If applicable, patients swallowed the CE by themselves. After the indicator light on the top of the recorder turned green, the recorder was connected to the image workstation to confirm that the capsule had entered the stomach. During the examination period, children were allowed to walk and keep away from the MRI room. Generally, CE could reach the small intestine 1 hour later. Metoclopramide was injected intramuscularly to promote gastric emptying if it did not enter the small intestine after 2 hours. The patients fasted within 4 hours after the capsule entered the small intestine, and only water was allowed.

If the CE did not reach the small intestine within 3 hours after swallowing, or if patients could not swallow the CE, the CE would be transported through painless gastroscopy under general anesthesia, which was referred to as gastroscopy-assisted CE. Before the operation, patients signed informed consent for anesthesia. The capsule was delivered to the descending segment of the duodenum with a foreign body net pocket or a snare. When the indicator light of the recorder turned green, the gastroscope and accessories were retrieved. The whole procedure was completed together by an endoscopist and an anesthesiologist. The recorder was removed after the CE reached the colon or was excreted, or the inspection time reached 12 hours. Then, images were uploaded to the image workstation and analyzed by 2 specific specialists. The final report was submitted after a discussion. Children and their parents were instructed to observe the stool to confirm whether the capsule was excreted.

### Observation Items

The following items were observed: (1) the completion rate of the entire small bowel examination and the related complications; (2) the potential risk factors for the incidence of gastroscopy-assisted CE, including gender, age, height, body weight, body mass index, chief complaint, and intestinal preparation medications; and (3) comparing the SBTT in various conditions with the corresponding control: distinct bowel preparation medications, use of metoclopramide, delivering the capsule under general anesthesia, and the existence of lesions in the small intestine.

### Statistical Analysis

Statistical Package for the Social Sciences (SPSS) 26.0 (IBM Corp.; Armonk, NY, USA) was used for statistical analysis. Potential risk factors were analyzed by univariate analysis, subsequently followed by multivariate regression analysis. One-way analysis of variance (ANOVA) was used to compare the means of groups. *P* < .05 was considered as statistical significance.

## Results

### General Information of Patients

**Figure 1.**
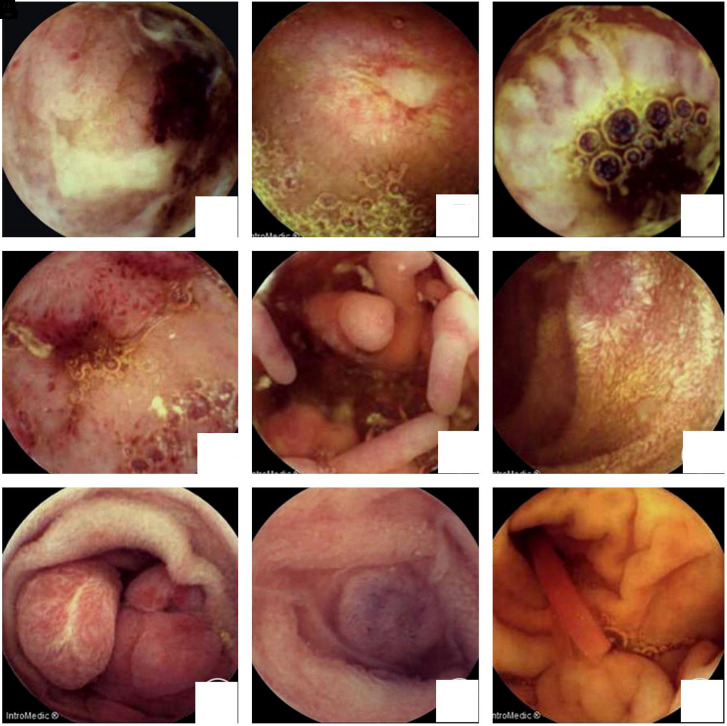
Images of capsule endoscopy. (A) Graft-versus-host disease (GVHD) after transplantation for thalassemia (ileal ulcer and bleeding). (B) Crohn’s disease (jejunum ulcer). (C) Ileal ulcer. (D) Congestion and erosion of the terminal ileum. (E) Crohn’s disease (proliferative lesion of the ileum). (F) Lymphangiectasia of the jejunum. (G) Jejunal polyps. (H) Blue rubber bleb nevus syndrome (vascular malformation). (I) Foreign object in the jejunum (plastic rod).

The general information of patients was shown in Table 1. As shown in Figure 1, the results from images of CE showed that the most common lesions were ulcers (31 cases, Figure 1A-C) and erosion (12 cases, Figure 1D), proliferative lesions (Figure 1E), lymphangiectasia (Figure 1F), polyps (Figure 1G), vascular malformation (Figure 1H), and submucosal bulges with foreign objects (Figure 1I), respectively.

### The Completion Rate of Entire Small Bowel Examination and the Relevant Complications

Further analysis showed that the CE passed through the ileocecal valve within the capsule battery working hours with the completion rate of the entire small intestine examination at 82% (196 out of 239 cases), and 3 children (1.3%) were subjected to capsule retention. Among them, CE remained in the ileum in 1 case diagnosed with Crohn’s disease, which was excreted 2 months after receiving enteral nutrition and infliximab, while another 2 patients were diagnosed with Crohn’s disease and cryptogenic multifocal ulcerative narrow enteritis, respectively. After the standard treatments, the clinical symptoms of these 2 children significantly improved. Up to now, the CE is still retained in the small bowel 6-8 months after placement. However, despite no clinical manifestations of intestinal obstruction being observed, such as abdominal distension and vomiting, we still continued to follow-up.

### Risk Factors for Incidence of Gastroscope-Assisted Placement of Capsule Endoscopy

As shown in Table 2, univariate logistic regression analysis showed that the incidence of gastroscope-assisted CE was associated with the age, height, body weight, and body mass index of patients (*P *< .05). while no significant difference was obtained in the analysis parameter of gender, chief complaint, or intestinal preparation medication (*P *> .05). Importantly, the further multivariate logistic regression analysis showed that the only age was a risk factor for the incidence of gastroscope-assisted CE [odds ratio 0.596% (CI, 0.436-0.813), *P *< .05] (Table 3).

### Effects of Intestinal Preparation Medications, Administration of Metoclopramide, General Anesthesia, and Small Bowel Disease on Small Bowel Transit Time of Capsule Endoscopy

As shown in Table 4, 1-way ANOVA was performed to compare the effect of relevant factors on SBTT. The results showed that general anesthesia for delivery of capsule significantly prolonged SBTT, which was statistically significant compared with patients without general anesthesia (*F *= 44.21, *P *< .01), while the other factors, such as intestinal preparation medications, metoclopramide, and small intestinal lesions, have not drastically affected SBTT (*P *< .05).

## Discussion

Capsule endoscopy was developed by Given Company (Israel) in 2001, providing a safe and non-invasive technology for visualization of the small intestine, which has been approved by the Food and Drug Administration (FDA) of the United States for children aged 10-18 years in 2004. Both CE and patency capsule were approved for children over the age of 2 years in 2009.^6^ In China, CE was approved for clinical application in 2002 and first applied to children by Ge et al.^7^

The Chinese guidelines for CE application have mainly recommended CE for obscure gastrointestinal bleeding, iron-deficiency anemia, suspected Crohn’s disease, suspected intestinal tumors, and monitoring of the therapeutic effects of Crohn’s disease patients and the progress of small-bowel polyposis syndrome.^5^ Similarly, the Spanish Society for Pediatric Gastroenterology guidelines also addressed that CE is mainly applied in children with Crohn’s disease, gastrointestinal bleeding, Peutz-Jeghers (P-J) syndrome, and suspected small-bowel diseases.^2^ In this study, the objective primarily induce a large number of patients involved in a variety of chronic abdominal pains, chronic diarrhea, gastrointestinal bleeding, and inflammatory bowel disease and a small number of patients suffered from anemia with unknown origin and P-J syndrome or manifested signs of repeated vomiting and abdominal distention.

Previous studies have shown that the minimum age and body weight of children who underwent CE was 8 months^8^ and 7.9 kg^9^ respectively, without capsule retention. A multi-center study reported that it is safe for children older than 1.5 years to perform CE. The minimum age of patients who could swallow the capsule was 4 years old; The gastroscope-assisted CE could be performed if the capsule ingestion was unavailable,^10,11^ suggesting that CE is highly safe in children with a wide range of ages. In our study, the minimum age of patients who received gastroscope-assisted CE was 2 years and 4 months, and the minimum body weight was 10.0 kg. We also found that the minimum age of patients who could swallow the capsule was 4 years. For those who could not cooperate with self-swallowing, or if the capsule failed to reach the small bowel 3 hours after swallowing, gastroscope was performed to deliver the CE into the small intestine under general anesthesia, with slight damage to the throat, esophagus, stomach, and duodenum.

A meta-analysis of CE in 740 children revealed that the completion rate of entire small intestine examination was 86.2%,^12^ which was slightly higher than that in our study (82%). The discrepancy between the 2 studies might be associated with the different incident rates of gastroscopy-assisted CE. Capsule retention, the most common complication of CE, was defined that as the condition when the capsule stayed in the gastrointestinal tract more than 2 weeks after examination. Aspiration of the CE into the airway is rare.^5^ Previous studies^12-14^ suggested that the capsule retention rate for CE was approximately 1%-2.6%, and most patients excreted the capsule without surgical interventions. Several factors contribute to capsule retention, including small-bowel bleeding and Crohn’s disease. It was reported that the capsule retention rate of patients with small-bowel bleeding, suspected or known Crohn’s disease was approximately 2%, 4%, and 8%, respectively.^15^ The retention rate could be significantly reduced if the small intestine conditions were evaluated in advance using patency capsule^16,17^ or MRI.^18,19^ According to the intelligent chromo capsule endoscope (ICCE) consensus, immediate surgery or endoscopic intervention is not recommended for the treatment of capsule retention if the patient is asymptomatic; the maximum retention time was up to 2.5 years.^20^ The capsule should be taken out through traditional endoscopy or surgery when the symptom of intestinal obstruction is observed, otherwise, the capsule could be excreted after patients were given drugs to eliminate the intestinal edema.^21,22^ Besides this, some researchers failed to get significant outcomes when applying paraffin oil to induce capsule elimination.^23^ However, there was no available report about whether paraffin oil would benefit pediatric patients suffering from capsule retention. In this study, in addition to 2 patients with the retained CE in the bowel after following up for 6-8 months, we found that the retention rate of capsules in 239 patients was 1.3%, which was comparable to other studies.^12,14^ Upon this, we highly recommend performing MRI or patency capsule to evaluate the intestinal conditions to decrease the risk of capsule retention.

Seidman and Dirks^24^ reported that the major factor affecting capsule swallow was the body weight but not the age of pediatric patients, and it was feasible for children over 16 kg to swallow the capsule. Burgess et al^25^ found that children’s age and body weight were significantly lower than those in the gastroscope-assisted CE group than in the self-swallowing group. In this study, the multivariate regression analysis results showed that age was a critical risk factor for the incidence of gastroscope-assisted CE but not height, body weight, body mass index, chief complaint, and bowel cleansing drugs.

In line with the studies,^9,25^ we also found that the SBTT was significantly increased in patients who underwent gastroscope-assisted CE compared with those who self-swallow the capsule. In general, gastroscopy is performed under general anesthesia. Inhalation of sevoflurane or intravenous injection of propofol is conventional. Both sevoflurane and propofol could prolong SBTT during the operation of endoscopy.^26,27^ Sevoflurane significantly slowed gastrointestinal motility.^27^ Besides, laying in bed for several hours after general anesthesia also contributes to the prolonging of SBTT. The increase of SBTT leads to a longer residence time of CE in the small intestine and lower the completion rate of entire small intestine examination. In the clinical practice, polyethylene glycol and simethicone are used to improve the bowel cleanliness, while neither of them changes the process of CE.^6^ In this study, polyethylene glycol or mannitol was used as an intestinal preparation drug. Both drugs induced favorable outcomes of cleanliness while having no effect on SBTT. Several drugs, such as domperidone metoclopramide, and mosapride, were shown to short SBTT in adults and increase the completion rate of entire small intestinal examination.^28-30^ However, our data suggested that no significant effect on the SBTT of pediatric patients were obtained in patients with metoclopramide treatment, which might be attributed to the limited cases administered this drug in this study. Further work is required to address this in the future. In addition, further results showed lesions of the small intestine did not significantly change the SBTT in children, which is not in line with that reported that gastrointestinal bleeding in adult patients slightly prolonged SBTT.^31^ There is no statistics on how many patients have not been diagnosed or may be misdiagnosed, which is a limitation of the present study. In clinical practice, after performing gastroscopy, abdominal B-ultrasound or CT, if it is suspected that there is a problem in the small intestine or that the lesion of the small intestine mucosa cannot be ruled out, CE will generally be performed. Clinically, most family members are more cooperative.

In fact, most of the children are not clearly diagnosed. In this study, gastroscopy-assisted CE was performed to understand whether there is intestinal mucosal damage. However, before performing CE, it is necessary to assess whether the child’s intestinal tract has the possibility of obstruction, such as abdominal distension and vomiting, and whether defecation is smooth. Capsule endoscopy can only be performed when abdominal B-ultrasound, abdominal x-ray, or enterography are performed to eliminate intestinal obstruction. If there is a risk of intestinal obstruction and stricture, CE is not recommended. In all the cases in this article, 1 child failed to discharge spontaneously after swallowing the capsule, and the intestinal stricture improved after medication, and then discharged smoothly.

In conclusion, CE is safe and effective for intestinal examination with few complications in children. The risk of capsule detention should be systematically evaluated before the operation. This study revealed that age is a critical risk factor for the incidence of gastroscope-assisted CE. Moreover, gastroscope-assisted CE significantly prolongs the SBTT and reduces the completion rate of the entire small-bowel examination, which might be associated with general anesthesia and postoperative bed rest for several hours in pediatric patients.

## Figures and Tables

**Table 1.** Patient Information and Details from Capsule Endoscopy Examination

Characteristics	Values
Patients, n (male : female)	239 (148 : 91)
Median age, years (range)	11.3 (2.3-17)
Median height, cm (range)	139 (85-175)
Median weight, kg (range)	30.45 (10-60)
Median BMI, kg/m^2^ (range)	15.28 (10.2-26)
The chief complaints	
Chronic abdominal pain, n (%)	147 (61.5)
Gastrointestinal bleeding, n (%)	29 (12.1)
Chronic diarrhea, n (%)	15 (6.3)
Inflammatory bowel disease, n (%)	30 (12.6)
Others (unexplained anemia, P-J syndrome, vomiting, and abdominal distension), n (%)	18 (7.5)
Gastroscopy-assisted CE (yes : no), n (%)	51 (21.3) : 188 (78.7)
Completion of total small bowel (yes : no), n (%)	196 (82) :43 (18)
Positive findings under CE (yes : no), n (%)	88 (36.8) :151 (63.2)
The extent of the lesion	
Jejunum only, n (%)	10 (11.4)
Ileum only, n (%)	42 (47.7)
Both jejunum and ileum, n (%)	36 (40.9)

BMI, body mass index.

**Table 2.** Univariate Logistic Regression Analysis of Risk Factors for the Incidence of Gastroscope-Assisted Placement of Capsule Endoscopy

Variables	*B*	SE	Wals	*P*	OR	95% CI	
						Lower	Upper
Gender	−0.214	0.336	0.403	.525	0.808	0.418	1.562
Age	−0.651	0.101	41.579	.000	0.521	0.428	0.635
Height	−0.084	0.014	37.567	.000	0.920	0.895	0.945
Body weight	−0.170	0.030	32.323	.000	0.843	0.795	0.894
BMI	−0.293	0.082	12.751	.000	0.746	0.635	0.876
Chronic abdominal pain	0.192	0.336	0.325	.569	1.211	0.627	2.341
Gastrointestinal bleeding	−0.777	0.441	3.102	.078	0.460	0.194	1.092
Chronic diarrhea	−0.484	0.609	0.633	.426	0.616	0.187	2.032
Inflammatory bowel disease	0.817	0.633	1.668	.197	2.263	0.655	7.821
Others	0.160	0.655	0.059	.807	1.173	0.325	4.238
Bowel preparation medications	−0.355	0.407	0.763	.382	0.701	0.316	1.556

BMI, body mass index; OR, odds ratio.

**Table 3.** Multivariate Logistic Regression Analysis for Factors Associated with the Incidence of Gastroscope-Assisted Placement of Capsule Endoscopy

Variables	*B*	SE	Wals	*P *	OR	95% CI	
						Lower	Upper
Age	−0.518	0.159	10.636	.001	0.596	0.436	0.813
Height	−0.003	0.082	0.001	.974	0.997	0.85	1.17
Body weight	−0.045	0.197	0.053	.819	0.956	0.65	1.405
BMI	−0.021	0.342	0.004	.951	0.979	0.501	1.915

BMI, body mass index; OR, odds ratio.

**Table 4.** Analysis of Factors Affecting SBTT

	Variables	n	SBTT (minutes)	*F*	*P*
General anesthesia	No	173	280.05 ± 129.97	44.21	.00
	Yes	23	475.95 ± 152.73		
Intestinal lesions	No	122	298.07 ± 148.01	0.37	.54
	Yes	74	311.23 ± 145.19		
Bowel cleansing drug	Mannitol	50	333.11 ± 155.94	2.85	.09
	Polyethylene glycol	146	292.74 ± 142.52		
Gastrointestinal prokinetic drug	No	188	305.70 ± 147.99	1.52	.22
	Yes	8	240.41 ± 99.60		

SBTT, small bowel transit time.
